# Exercise Interventions Combined With Dietary Supplements in Type 2 Diabetes Mellitus Patients—A Systematic Review of Relevant Health Outcomes

**DOI:** 10.3389/fnut.2022.817724

**Published:** 2022-03-09

**Authors:** Frederike Maria Meuffels, Eduard Isenmann, Malte Strube, Alessio Lesch, Max Oberste, Christian Brinkmann

**Affiliations:** ^1^Department of Preventive and Rehabilitative Sport Medicine, Institute of Cardiovascular Research and Sport Medicine, German Sport University Cologne, Cologne, Germany; ^2^IST University of Applied Sciences, Düsseldorf, Germany; ^3^Department of Molecular and Cellular Sport Medicine, Institute of Cardiovascular Research and Sport Medicine, German Sport University Cologne, Cologne, Germany; ^4^Institute of Medical Statistics and Computational Biology (IMSB), Faculty of Medicine and University Hospital Cologne, University of Cologne, Cologne, Germany

**Keywords:** dietary supplements, diabetes mellitus, physical activity, exercise, training, sports

## Abstract

**Introduction:**

Physical training can improve several health variables in patients with type 2 diabetes mellitus (T2DM). A growing body of studies also finds a positive influence of dietary supplement (DS) intake. The aim of this review is to shed light on the possible effects of training interventions combined with DS intake in T2DM patients.

**Methods:**

A systematic search was performed following the Preferred Reporting Items for Systematic Reviews and Meta-Analyses (PRISMA) guidelines in the PubMed and BISp Surf databases. Inclusion criteria were defined using the Patient-Intervention-Comparison-Outcome (PICO) scheme. The Physiotherapy Evidence Database (PEDro) scale was used for quality assessment and risk of bias analysis.

**Results:**

Ten controlled interventional studies with a total number of 643 subjects met the inclusion criteria. These studies investigated the effects of (a) vitamin D (VD), (b) VD + whey protein, (c) polyphenol containing antioxidant capsules, (d) creatine, (e) L-arginine, (f) leucine-rich amino acids, and (g) broccoli sprouts powder. Eight studies investigated effects on one or more of the following health outcomes: body mass index, fat mass, insulin resistance, glycemic control, lipid profile, oxidative stress/antioxidative capacity and/or inflammatory markers/molecules. Five of the studies show clear superior effects of physical training combined with DS intake (supplements a, b, c, e) on some of these variables compared with training only. However, one study indicates that VD intake might attenuate the training effects on triglyceride levels. Another study found that training + VD + whey protein intake increased tumor necrosis factor-α levels in T2DM patients. The effects of training combined with DS intake on renal function (supplement d) or incretin metabolism (supplement a) were investigated in two further studies. These studies do not show any additional effects of DS intake. The quality of the majority of the studies was high.

**Conclusion:**

DS intake can potentially increase the benefits of physical training for specific health outcomes in T2DM patients. However, negative effects can also be observed. Possible cellular and molecular mechanisms behind potential synergistic or divergent effects of exercise training and DS use in T2DM should be explored in detail in future studies for the development of safe recommendations.

## Introduction

Worldwide, ~462 million people are affected by type 2 diabetes mellitus (T2DM), of which over one million die annually (1). T2DM and secondary complications of T2DM are preventable in most cases by maintaining a healthy diet, engaging in regular physical activity, maintaining a normal body weight, and avoiding tobacco use (2). Lifestyle modifications have been shown to reduce the risk of developing T2DM by ~40–70% (3). Hence, lifestyle management of patients is a crucial factor in the prevention of T2DM.

Regular physical activity does not only help prevent the onset of T2DM but can also improve T2DM variables, such as body mass index (BMI), glycemic control and variability, insulin sensitivity, the lipid profile, oxidative stress/antioxidative capacity and/or chronic inflammation (4, 5). Therefore, increasing daily physical activity and structured exercise programs are recommended in the therapy of T2DM (5).

A potential benefit related to these outcomes has also been suggested for the intake of various dietary supplements (DS) in T2DM [for excellent reviews on their effects and mechanisms of action, see (6–8)]. These dietary supplements include, for example, vitamins such as vitamin C, folic acid or vitamin D (9–11), minerals such as chromium or zinc (12, 13), dietary fiber (14), amino acids like L-arginine (15), complex fruit, vegetable—or plant extracts such as pomegranate, broccoli sprouts or tea extracts (16–18).

Given the benefits of regular physical exercise in T2DM patients (5) and the increasing interest in DS intake (6–8), the aim of this systematic review is to shed light on the effects of physical exercise programs combined with DS intake on health outcomes in T2DM patients, primarily on BMI, body fat mass, glycemic control, insulin sensitivity, the lipid profile, oxidative stress/antioxidative capacity and/or inflammatory markers/molecules. These variables may predict diabetes complications, adverse cardiovascular events and mortality (19–24). To our knowledge, this is the first systematic review on the effects of physical training combined with DS intake on health outcomes in T2DM patients. The findings could help optimize the efficacy of training interventions and reduce the progression of the disease and its complications to the highest extent possible.

## Methods

### Literature Search

A systematic literature review was conducted based on the PRISMA (Preferred Reporting Items for Systematic Reviews and Meta-Analyses) guidelines (25). Two researchers (FM, CB) scanned PubMed/MEDLINE and the BISp Surf database (database of the German Federal Institute of Sports Sciences including the databases of DOAJ Articles, CiteSeerX, BioMed Central, MDPI Open Access Publishing, Zenodo) for eligible studies up to 29 December, 2021. The search was not limited to a start date. The detailed search strings are provided in Table 1.

**Table 1:** Search strategy.

**Databases**	**Search strategy**
PubMed/MEDLINE	(“diabetes” OR “diabetic” OR “T2DM” OR “T2D” OR “insulin resistance” OR “insulin-resistant”) AND (“training” OR “exercise” OR “physical activity”) AND (“supplement” OR “vitamin” OR “antioxidant”) AND (“control group” OR “controlled” or “placebo”) No filters
BISp Surf Portal of the Federal Institute of Sports Sciences Germany	(diabetes OR diabetic OR T2DM OR T2D OR insulin resistance OR insulin-resistant) AND (training OR exercise OR physical activity) AND (supplement OR vitamin OR antioxidant) AND (control group OR controlled OR placebo) Allow further data sources, exclude PubMed

### Eligibility Criteria

Only articles published in peer-reviewed scientific journals were included. Studies had to be available as full texts in the English language. Additional inclusion criteria were defined using the PICO (Patient-Intervention-Comparison-Outcome) scheme (26). (1) For inclusion, study participants (of all genders) had to have been diagnosed with T2DM. (2) Furthermore, a training intervention (chronic exercise, all types of training) with the intake of DS was required. (3) Only controlled trials were included. The studies' control groups had to also consist of patients diagnosed with T2DM. The participants in the control groups had to participate in the training intervention without having consuming DS. (4) The studies were required to report on health outcomes relevant for patients with T2DM. Studies were included in further analyses, if they reported on one of the following outcome variables: BMI, body fat mass, glycemic control, insulin sensitivity, the lipid profile, oxidative stress/antioxidative capacity and/or inflammatory markers/molecules. However, studies were also included if they did not consider one of these variables, but if their primary outcome variable was rated as relevant for the health of T2DM patients by the researchers at a joint meeting (FM, EI, CB). Studies had to include adequate statistical information (at least mean values, measure of dispersion, statistical test(s) used, significance level).

### Study Selection

In a first step, any duplicates were removed. Then, the titles and abstracts of articles were checked for relevance by two researchers (FM, CB). They subsequently, independently from each other, reviewed the full texts of potentially eligible articles. Any disagreements were discussed with a third researcher (EI) until consensus was reached.

### Data Extraction

The authors, publication date, number, age and sex of included T2DM patients, dietary supplement(s) and dosing regime, information on the exercise intervention (training type, training frequency), duration of the program, information on the control group(s) (program, number of participants), list of outcome variables (according to Section Eligibility Criteria) and main statistical results (changes from baseline within and/or between groups) were extracted from the studies.

### Quality Assessment and Risk of Bias

Two researchers (FM and EI) assessed the quality of included studies using the PEDro (Physiotherapy Evidence Database) scale (27). Any disagreements were discussed with a third researcher (CB) until consensus was reached. The PEDro score is a valid measure of the methodological quality of clinical trials. The checklist contains 11 items [for external validity (item 1), internal validity (items 2–9), and statistical reporting (items 10–11)]. The final PEDro score is achieved by adding the ratings of items 2 to 11 (each item: yes = 1 or no = 0) for a total score between 0 and 10. The fulfillment of <4 criteria is considered “poor”, 4–5 is considered “fair”, 6–8 is considered “good” and 9–10 is considered “excellent” (27).

## Results

**Figure 1:**
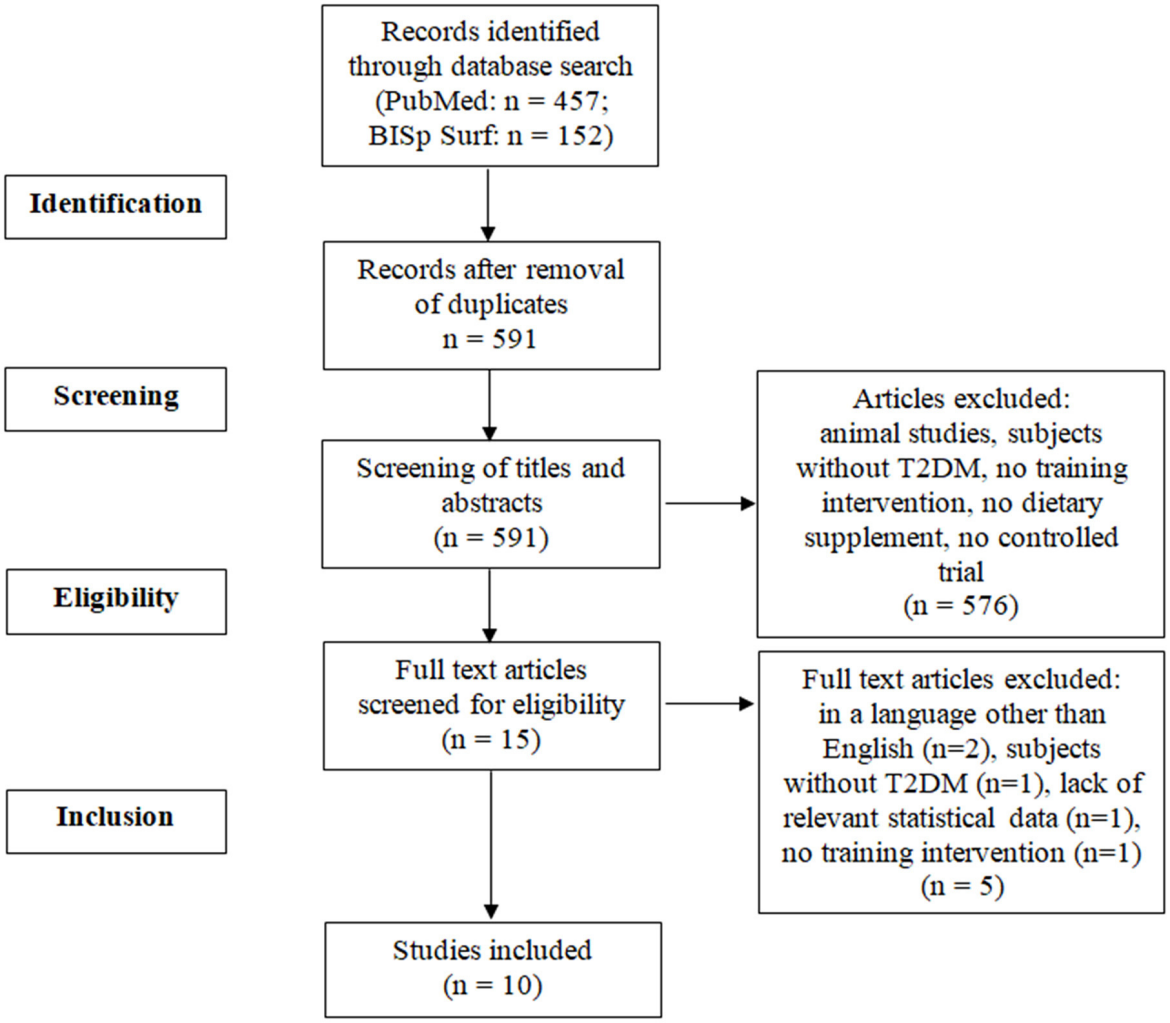
PRISMA flow diagram. T2DM, type 2 diabetes mellitus.

The selection process is presented in the flow diagram (Figure 1). Ten studies met the inclusion criteria (28–37). The studies were published between 2006 and 2021. In total, 643 T2DM patients were included. The detailed characteristics and main outcomes of the studies are presented in Table 2. The PEDro scale ratings are presented in Table 3.

**Table 2:** Detailed characteristics and outcomes of the studies.

**Study**	**T2DM subjects**	**Dietary supplement**	**Exercise intervention**	**Duration (weeks)**	**Groups**	**Selected important outcome variables (BMI, fat mass, glycemic control, insulin sensitivity, lipid profile, oxidative stress/antioxidative capacity and inflammatory markers/molecules, other variable(s) if explicitly defined as primary outcome variable(s) in the studies)**	**Main results (significant changes that differed among the groups)**
Dadrass et al. (28)	*n* = 48 Men Vitamin D deficit Age: 40–65 years	Vitamin D (VD) (50.000 IU per 2 weeks for 3 months)	Resistance training (RT) 3 times per week	12	1. VD+RT; *n* = 12 2. Placebo + RT; *n* = 12 3. VD; *n* = 12 4. Placebo; *n* = 12	• BMI • HbA1c • HOMA-IR index • LDL • HDL • TG • IL-6 • TNF-α • CRP	Repeated measures ANOVA results and *post-hoc* tests results to determine statistical significance for changes over time within groups: - BMI and TNF-α levels significantly decreased only in the VD + RT group and placebo + RT group (significant time × group interaction effect). - HbA1c and HOMA-IR index values significantly decreased only in the VD+RT group and VD group (significant time × group interaction effect). - TG levels significantly decreased only in the RT+placebo group (significant time × group interaction effect). → Some health outcomes were positively affected when RT and VD were combined (compared with training only or VD only) → It seems that many health variables were improved in the VD+RT group as much as possible → Vitamin D supplementation may abolish the training effects on TG levels
Fenercioglu et al. (29)	*n* = 114 Men (43) and women (71) Age: 40–65 years Newly diagnosed with T2DM	Polyphenol containing antioxidant capsule (AO) (1 per day; containing 500 mg pomegranate extract, 300 mg green tea extract and 60 g vitamin C)	Aerobic endurance exercise training (AE) 150 min a week + standard diet (D) of 1,500 kilocalories per day. Diet started before the first measurement to build standardization of oxidative stress among the participants	12	1. AO + AE + D; *n* = 56 2. Placebo + AE + D; n= 58	• HbA1c • LDL • HDL • AOC • GSH • MDA	Paired t-tests/Wilcoxon signed-rank tests to determine statistical significance of changes over time in each group. Mann-Whitney tests to determine statistical significance of differences in pre- post change values between groups: - LDL levels significantly decreased, and HDL levels significantly increased only in the AO+AE+D group. Significant difference between groups for pre-post change value comparison. - MDA levels were significantly reduced in the AO+AE+D group, but did not change in the placebo + AE + D group. Significant difference between groups for pre-post change value comparison. - AOC significantly increased in the AO + AE + D group, but did not change in the placebo + AE + D group. Significant difference between groups for pre-post change value comparison. - GSH levels increased significantly in both groups, but to a significant higher extent in the AO+AE+D group. → Some health outcomes were positively affected when AE and AO were combined (compared with training only) → There were no negative effects of the supplementation on any outcomes, nor did AO supplementation abolish or diminish any training effects
Gualano et al. (30)	*n* = 25 Men (9) and women (16) Age: 1. 58 ± 5 years 2. 56 ± 8 years	Creatine monohydrate (CR) (5 g/day; powder in water during lunch)	Aerobic endurance exercise training (AE) combined with resistance training (RT) 3 times per week	12	1. CR + AE/RT: *n* = 13 2. Placebo + AE/RT: *n* = 12	• ^51^Cr-EDTA clearance (renal function) (primary outcome)	Mixed model for repeated measures with fixed effects for group and time (pre vs. post) and random effect for subject + *post-hoc* tests results: - No significant results. → There were no negative effects of the supplementation on any outcomes, nor did CR supplementation abolish or diminish any training effects
Kim et al. (31)	*n* = 52 Women Vitamin D deficit Age: 1. 70 ± 1 years 2. 69 ± 1 years 3. 73 ± 2 years 4. 70 ± 1 years	Vitamin D (VD) (1.200 IU per day for 3 months)	Circuit training (CT): Aerobic endurance exercises were alternated with resistance exercises 3 times per week in weeks 1–6 and 4 times per week in weeks 7–12	12	1. VD + CT; *n* = 15 2. Placebo + CT; *n* = 11 3. VD; *n* = 13 4. Control (placebo); *n* = 13	• BMI • HOMA-IR index • LDL • HDL • TG	Repeated measures ANOVA results and *post-hoc* tests results to determine statistical significance for changes over time within groups: - LDL levels significantly decreased only in the VD+CT and placebo + CT group (significant time × group interaction effect).- HDL levels significantly increased only in the VD+CT group and placebo + CT group and significantly decreased in the VD and control group (significant time × group interaction effect). - TG levels significantly decreased only in the VD+CT group (significant time × group interaction effect). → Some health outcomes were positively affected when CT and VD were combined (compared with training only or VD only) → VD intake had negative effects on HDL levels, but did not abolish or diminish any training effects
Lucotti et al. (32)	*n* = 33 Men (8) and women (25) Age: 56 ± 1 years Insulin resistant	L-arginine (LA) (8.3 g per day for 21 days)	Aerobic endurance exercise training (AE) combined with resistance training (RT) 45 min twice a day for 5 days per week for 3 weeks + hypocaloric diet (D) 1,000 kcal/day	3	1. LA + AE/RT + D; *n* = 16 2. Placebo + AE/RT + D; *n* = 17	• FM • HOMA-IR index • LDL • HDL • TG • SOD • Adiponectin	Repeated measures ANOVA results: - A significant time × group interaction effect was detected for FM, HOMA-IR index, LDL levels, which was based on a greater decrease in the LA + AE/RT + D group compared to the placebo + AE/RT + D group. - A significant time × group interaction effect was detected for SOD levels, which was based on an increase in the LA + AE/RT + D group and a decrease in the placebo + AE/RT + D group. - A significant time × group interaction effect was detected for adiponectin level, which was based on an increase in the LA + AE/RT + D group, while the mean did not change over time in the placebo +AE/RT + D group.
							→ Some health outcomes were positively affected when AE/RT+D and LA were combined (compared with training only) → It seems that many health variables were improved in the LA + AE/RT + D group as much as possible → There were no negative effects of the supplementation on any outcomes nor did LA supplementation abolish or diminish any training effects
Miller et al. (33)	*n* = 198 Men (126) and women (72) (176 and 167 participants completed the 12- and 24-week assessment) Age:50–75 years 1. 61 ± 6 years 2. 62 ± 6 years	Vitamin D (VD) (2.000 IU/day) + whey protein (WP) (20 g protein/day & 20 g within 2 h after RT).	Resistance training (RT) Twice a week in the first 8 weeks, 3 times per week in the last 16 weeks	24	1. VD + WP + RT; *n* = 98/*n* = 87 2. RT; *n* = 100/*n* = 80	• FM • HbA1c • HOMA2- IR index • IL-6 • IL-8 • IL-10 • TNF-α • hs-CRP • Adiponectin • Resistin	GLMM for repeated measures results with fixed effects for group, time (pre vs. post) and time × group interaction (adjusted for age, sex and diabetes management) at 12 and 24 weeks, as well as random effect for subject with estimated marginal means for time × group interaction: - IL-10 levels significantly increased in the VD + WP + RT group at 12 and at 24 weeks. The IL-10 levels significantly decreased in the control training group at 24 weeks. - TNF-α levels significantly increased in the VD +W P + RT group at 24 weeks and did not change in the control training group. → IL-10 levels were positively affected when RT and VD+WP were combined (compared with training only) → Most health variables were similarly affected through the training interventions → VD+WP intake negatively affects TNF-α level training outcomes
Rahimi et al. (34)	*n* = 48 Men Age: 40–60 years	Vitamin D (VD) (50.000 IU once per week)	Aerobic endurance exercise training (AE) 3 times per week	8	1. Aerobic exercise (AE) 2. VD 3. AE + VD 4. Control	• GLP-1 (primary outcome) • DPP-4 (primary outcome)	ANCOVA results with baseline value as covariate and between-subject factor group, as well as *post-hoc* comparisons: - GLP-1 levels were significantly higher and DPP-4 levels were significantly lower in the training groups (AE or AE+VD) post-training compared to the VD or control group. → Some health outcomes were positively affected when AE and VD were combined (compared with VD only) → Health variables were similarly affected through the training interventions → There were no negative effects of the supplementation on any outcomes, nor did VD supplementation abolish or diminish any training effects
Saeidi et al. (35)	*n* = 44 Men Age: 40–60 years	Broccoli sprouts powder (B) 10 g per day	Aerobic endurance exercise training (AE) combined with resistance training (RT) 3 times per week	12	1. B + AE/RT; *n* = 11 2. Placebo + E/RT; *n* = 11 3. B; *n* = 11 4. Placebo; *n* = 11	• BMI • FM • HOMA-IR index • IL-6 • TNF-α • hs-CRP • Dectin-1 • LDL • HDL • TG	Repeated measures ANOVA results and *post-hoc* tests results to determine statistical significance for changes over time within groups. - BMI and FM significantly decreased only in the B+AE/RT group and P+AE/RT group (significant time × group interaction effect). - HOMA-IR index values significantly decreased and HDL levels significantly increased only in the training groups (B+AE/RT or placebo + AE/RT) and in the B group (significant time × group interaction effect) with greater improvements in the training groups - hs-CRP, IL-6, TNF-α and dectin-1 levels significantly decreased only in the training groups (B+AE/RT or placebo + AE/RT) and in the B group (significant time × group interaction effect). - LDL levels significantly decreased only in the training groups (B+AE/RT or placebo + AE/RT) and in the B group (significant time × group interaction effect) with a greater decrease in the B+AE/RT group compared with the B group - TG levels significantly increased in the B group and significantly decreased in the training groups (B+AE/RT or placebo+AE/RT) (significant time × group interaction effect). → Some health outcomes were positively affected when AE/RT and B were combined (compared with B only) → B intake had negative effects on TG levels, but did not abolish or diminish any training effects
Singla et al. (36)	*n* = 28 Men (13) and women (5) Age: 25–50 years 1. 39 ± 8 years 2. 42 ± 7 years 3. 42 ± 7 years Vitamin D deficiency	Simvastatin (S) (40 mg per day) Vitamin D (VD) (60,000 IU once per week)	Aerobic endurance exercise training (AE) 5 times per week	12	1. S + VD-placebo + AE; n=9 2. S + VD + AE; *n* = 9 3. VD + S-placebo + AE; *n* = 10	• BMI • HbA1c • LDL • HDL • TG • VO_2peak_ (primary outcome) • Skeletal muscle citrate synthase activity (primary outcome)	Paired *t*-tests to determine statistical significance of changes over time within each group. Comparisons of differences in pre-post change values between groups were reported only for VO_2peak_ and citrate synthase activity: BMI decreased significantly only in the S + VD + AE group and in the VD + S-placebo + AE group. - LDL levels significantly decreased only in the S + VD-placebo + AE and S + VD + AE groups. - TG significantly decreased only in the VD + S-placebo + AE group. - VO_2peak_ significantly decreased in the S + VD-placebo + AE group and significantly increased in the VD + S-placebo + AE group. The changes from pre to post differed significantly between the S+VD placebo + AE group and the VD + S-placebo + AE group.
							→ BMI was positively affected when S+AE and VD were combined (compared with S+training only) → VD supplementation attenuated the negative effects of simvastatin on training outcomes (VO_2peak_) → There were no negative effects of the supplementation on any outcome
Yamamoto et al. (37)	*n* = 53 Men (28) and women (25) Age: 73 ± 2 years	Leucine-rich amino acids (L) (Amino L40; 1.2 g leucine, 1.8g other amino acids, twice a day)	Resistance training (RT) every day	48	1. L + RT; *n* = 18 2. RT; *n* = 18 3. Control; *n* = 17	• BMI • FM • HbA1c • Knee extension strength (primary outcome)	Paired *t*-tests to determine statistical significance of changes over time within each group. One-way ANOVA with between-subjects factor group on pre-post-change values: - Knee extension strength significantly increased only in the L+RT group. The change in muscle strength was significantly higher in the combined RT groups (L+RT, RT) compared to the control group. → Most health variables were similarly affected through the training interventions → One performance outcome was positively affected when L and RT were combined (compared with training only) → L supplementation did not abolish or diminish any training effects

*ANCOVA, analysis of covariance; ANOVA, analysis of variance; AOC, antioxidant capacity; BMI, body mass index; ^51^Cr-EDTA, chromium-51 labeled ethylenediamine tetraacetic acid (clearance: glomerular filtration rate); CRP, C-reactive protein; DPP 4, dipeptidyl peptidase-4; FM, fat mass; GLMM, generalized linear mixed model; GLP 1, glucagon like peptide-1; GSH, total glutathione; HbA1c, glycated hemoglobin; HDL, high-density lipoprotein; HOMA-IR, homeostasis model assessment for insulin resistance; hs-CRP, high sensitive C-reactive protein; IL-6,8,10, interleukin 6,8,10; LDL, low-density lipoprotein; MDA, plasma malondialdehyde; TG, triglycerides; SOD, superoxide dismutase; TNF-α, tumor necrosis factor alpha; VO_2peak_, peak oxygen consumption*.

**Table 3:** PEDro scale ratings.

**Study**	**Points**
Dadrass et al. (28)	10/10
Fenercioglu et al. (29)	10/10
Gualano et al. (30)	10/10
Kim et al. (31)	10/10
Lucotti et al. (32)	10/10
Miller et al. (33)	10/10
Rahimi et al. (34)	6/10
Saeidi et al. (35)	10/10
Singla et al. (36)	10/10
Yamamoto et al. (37)	7/10

### Vitamin D

Five of the eligible studies investigated the effect of vitamin D (VD) (28, 31, 33, 34, 36). Different dosages of VD supplements were taken over different time spans. Furthermore, different types of training interventions were included.

The effect of VD supplementation in combination with either resistance training (RT), aerobic endurance exercise training (AE) or combined resistance and endurance training on glycemic control and/or insulin resistance was investigated in four studies (28, 31, 33, 36). Glycated hemoglobin (HbA1c) and/or the homeostasis model assessment for insulin resistance (HOMA-IR) index and were analyzed.

In the study of Dadrass et al. (28), VD intake and RT were combined. HbA1c decreased significantly when VD intake and RT were combined. HbA1c decreased also in the group that took VD supplements only but it did not in the group that received a placebo+training. By contrast, the participants in the study of Miller et al. (33) performed RT and took VD supplements + whey protein (WP), but no significant effect of VD supplementation combined with regular physical exercise on HbA1c levels was found at the end of the intervention. There was also no significant change in the control group (RT-only group). The study of Singla et al. (36) examined the effects of VD supplementation combined with AE on the side effects of simvastatin. HbA1c levels did not change in any group.

In the studies of Dadrass et al. (28) and Miller et al. (33) HOMA-IR index (HOMA2-IR in Miller et al.'s study) decreased significantly in the VD+RT groups. Furthermore, HOMA-IR index also decreased significantly in the VD-only group in Dadrass et al.'s study (28). In the study of Kim et al. (31), the participants performed circuit training and consumed VD supplements. No significant effect on HOMA-IR index was observed (neither in the intervention nor in the control groups).

The lipid profile was monitored in three studies (28, 31, 36). The effects of regular physical exercise and VD on low density lipoprotein (LDL), high density lipoprotein (HDL) and triglycerides (TG) were investigated. In the study of Dadrass et al. (28), TG decreased significantly only in the RT + placebo group. No significant time^*^group effects were observed for LDL and HDL. By contrast, Kim et al. (31) were able to show a significant decrease of LDL and TG levels and a significant increase of HDL levels in the VD + CT group, and they showed a significant decrease of LDL and increase of HDL in the CT-only group. However, in the VD-only and placebo group, HDL levels decreased significantly. Singla et al. (36) showed a significant LDL decrease in the training groups (+simvastatin), independent of VD intake. TG levels only fell significantly for the VD+AE group (no simvastatin intake).

Pro- and/or anti-inflammatory markers/molecules, such as C-reactive protein (CRP), interleukin 6 (IL-6), interleukin 8 (IL-8), tumor necrosis factor alpha (TNF- α) or interleukin 10 (IL-10) were analyzed in two studies (28, 33). Dadrass et al. (28) found significant changes in TNF-α and IL-6 levels. TNF-α levels decreased significantly in the VD+RT and RT groups, and IL-6 decreased in all intervention groups (VD+RT, RT, VD). In the study of Miller et al. (33), anti-inflammatory IL-10, but also pro-inflammatory TNF-α, increased significantly when RT+VD+WP were combined. In the control group (RT only), IL-10 levels decreased significantly while TNF-α levels did not change.

Rahimi et al. (34) investigated the effects of VD supplementation combined with AE on glucagon-like peptide 1 (GLP-1) and dipeptidylpeptidase 4 (DPP-4). GLP-1 and DPP-4 have important roles in insulin secretion and control of blood glucose levels. GLP-1 can stimulate glucose-dependent insulin secretion and increase insulin sensitivity. The DPP-4 enzyme can reduce active GLP-1 (38). Statistical analyses revealed an increase in GLP-1 and a decrease in DPP-4 levels following training—independent of VD intake (34).

Overall, many health outcomes were positively affected when VD and physical training were combined. It was shown that many health variables were improved as much as possible with the combination of VD and regular exercise. By contrast, the study of Dadrass et al. (28) also shows that VD supplementation may attenuate the training effects on triglycerides. In the study of Kim et al. (31), LDL levels were negatively influenced by VD only. Miller et al. (33) report a negative influence of VD supplementation on TNF-α levels which increased following VD+WP+RT. It is also important to note that Singla et al. (36) found that VD supplementation minimizes the negative effects of simvastatin intake on peak oxygen consumption (VO_2peak_).

### Polyphenol Containing Antioxidant Capsules (Pomegranate Extract, Green Tea Extract, and Vitamin C)

Fenercioglu et al. (29) investigated the effects of a supplement containing pomegranate extract, green tea extract and vitamin C (AO) combined with an AE program. The control group received a placebo supplement and followed the AE program. In their study, they found that the antioxidant capacity (AOC) and HDL levels increased significantly, and LDL and plasma malondialdehyde (MDA) levels decreased significantly only in the AO + AE group. Glutathione levels increased significantly in both groups, but to a higher extent in the AO + AE group. In their study, many health outcomes were positively influenced by the supplementation of pomegranate extract, green tea extract and vitamin C in combination with AE. There were no negative effects of this DS on any health outcome, nor did this DS abolish or diminish any training effects.

### Creatine

Gualano et al. (30) tested the safety and influence of a creatine monohydrate supplementation combined with AE on the glomerular filtration rate of T2DM patients. T2DM patients are at risk of chronic kidney disease. According to the authors, case reports stated that creatine supplementation may induce kidney dysfunction. Therefore, the effects on T2DM patients were investigated in this study. To explore the possible effects of creatine on T2DM patients' kidney function, the glomerular filtration rate can be monitored. Gualano et al. did not detect any significant differences on renal function in comparison to the control group that received a placebo instead of creatine. The supplementation of creatine did not abolish or diminish any training effects. It was concluded that creatine supplementation combined with an AE program may be safe for T2DM patients with controlled hypertension, metabolic control and without chronic kidney disease.

### Amino Acids

The effects of amino acids were investigated in two studies (32, 37). The study of Lucotti et al. (32) aimed to investigate the effects of L-arginine (LA) supplementation, a hypocaloric diet, and physical exercise training (RT + AE) on the variables of the glucose and lipid profile. The control group received a placebo instead of LA. HOMA-IR improved more in the LA + RT + AE group. Superoxide dismutase (SOD) levels increased in the LA + RT + AE group and decreased in the control group. Also, adiponectin levels increased in the LA + RT + AE group but did not change in the control group.

Yamamoto et al. (37) examined the influence of leucine supplementation and RT on strength, body composition and glycemic control compared to RT only and a control group that neither received a placebo nor exercised. No significant differences in the glycemic variables such as HbA1 or in body composition between the groups were observed. Knee extension strength significantly increased only in the leucine + RT group.

### Broccoli Sprouts Powder

Saeidi et al. (35) investigated the effects of broccoli sprouts powder (B) in addition to physical training (combined AE/RT). The control groups received training + placebo, B only or placebo only. HOMA-IR index values significantly decreased and HDL levels significantly increased only in the training groups and B group with greater improvements in the training groups. LDL levels only significantly decreased in the training groups and B group with a greater decrease in the B + AE/RT group compared with the B group. hs-CRP, IL-6, TNF-α and dectin-1 levels only significantly decreased in the training groups and B group. It is also important to note that Saeidi et al. (35) found that TG levels significantly increased in the B group, while TG levels significantly decreased in the training groups (B + AE/RT or placebo + AE/RT).

## Discussion

The aim of this review is to shed light on the possible effects of training interventions combined with DS intake on relevant health variables in T2DM patients. It seeks to clarify whether T2DM patients who exercise regularly should take DS to maximize the training effects.

Compared with training only, positive effects of DS intake in combination with regular physical exercise on insulin resistance were observed in two studies (VD, LA) (28, 32), on glycemic control in one study (VD) (28), on the lipid profile (LDL, HDL or TG levels) in three studies (AO, VD, LA) (29, 31, 32), on oxidative stress/antioxidative capacity markers/molecules in two studies (AO, LA) (29, 32) and on inflammatory markers/molecules in two studies (LA, VD + WP) (32, 33).

In two studies, negative effects of DS intake were observed compared with training only (28, 33). In these studies, VD supplements were consumed.

The results suggest that VD supplementation may help increase insulin sensitivity and glycemic control in T2DM patients participating in a training intervention (28). It has been demonstrated in *in vivo* and *in vitro* studies that VD plays an important role in maintaining pancreatic ß-cell function, among others, by regulating calcium flux (39). Experimental studies have also shown that VD may help reduce the accumulation of advanced glycation end-products which have been linked to insulin resistance (39). Therefore, VD intake may have additional beneficial effects on insulin secretion and skeletal muscle insulin sensitivity, particularly in VD-deficient patients, compared with training only. Regarding possible additive effects of VD supplementation on triglyceride levels, training studies have shown divergent results (ranging from beneficial to negative effects) (28, 31). The menchanisms by which VD may lower cholesterol levels have been investigated in previous studies and include, for example, deactivation of sterol regulatory-element binding protein-2 (SREBP2) (40). However, Dadrass et al. (28) showed that VD+RT had no significant effect on TG levels, in contrast to RT alone. Nevertheless, even in the VD+RT group, the mean TG level was lower post-training compared with baseline pre-training. One possible explanation for the non-significant effect of VD+RT on TG levels could be the (non-significant) lower TG level in the VD + RT at baseline compared to that of the RT group. The small sample size must also be taken into account. On the other hand, it cannot be excluded that VD intake may attenuate the effects of training. Although the exact molecular mechanisms are unknown, possible reasons can be speculated. VD has been supposed to act as an antioxidant or at least to be involved in increasing the gene expression of antioxidants (41). It is currently debated whether and to what extent alteration in redox state by antioxidants ingestion might prevent training effects due to the fact that exercise-induced free radicals trigger signaling pathways to induce several biological adaptations to training (42). Regarding further effects of VD supplementation + training, another study found that VD+WP supplementation + RT may increase anti-inflammatory IL-10 (in contrast to a decrease with training only) (33). Surprisingly, VD + WP intake + RT also increased pro-inflammatory TNF-α levels (in contrast to no change with training only) (33). In this context, anti-inflammatory actions of VD have been demonstrated *in vitro*, and VD has been shown to suppress the activity of nuclear factor kappa B (NF-κB), an important pro-inflammatory transcription factor in immunomodulation (43).

In contrast to this finding is the increase in TNF-α levels, even considering that the change in body composition in the VD+WP intake + RT group (decrease in body fat mass) was similar to that of the control group (training only) and that adipose tissue is known to release a large amount of pro-inflammatory cytokines (44). Studies on cellular and molecular mechanisms are needed to explain this result.

Polyphenol containing AO capsules (pomegranate extract, green tea extract, vitamin C) showed several positive additional effects (increase in antioxidant molecules (glutathione levels)/capacity, reduction in oxidative stress (MDA levels), improvements in lipid profiles post-training). In addition to antioxidant vitamin C, pomegranates and tea have potent antioxidant properties due to their phenolic compounds (45). It is generally accepted that oxidative stress promotes T2DM and its complications (46). Therefore, reducing oxidative stress through training and/or dietary supplement intake is important to improve metabolic control in T2DM patients (46). Metabolic benefits could also interfere with lipid pathways and reduce dyslipidemia (47).

Positive additive effects have also been shown for LA. LA supplementation + physical training may help reduce body fat, increase insulin sensitivity, improve lipid profile, and increase antioxidant SOD and anti-inflammatory adiponectin levels in T2DM patients (32). In an animal study, LA was shown to increase the expression of key genes responsible for fatty acid and glucose metabolism (48). Furthermore, LA has been shown to decrease NF-κB levels (49).

Attention should also be paid to the potential of DS intake and regular physical exercise in buffering the side effects of medication. In the study of Singla et al. (36), VD reduced the side effects of statins (a significant decrease in VO_2peak_). Thus, DS may be particularly useful for T2DM patients in maintaining their physical fitness. However, underlying mechanisms are unknown and need to be explored in future studies.

Although some studies indicate that it might be beneficial to combine the intake of DS with regular physical exercise to maximize training effects, current research is insufficient to draw clear conclusions about the additive effects of physical exercise and DS in T2DM patients. Reasons for this are the low number of available studies, the studies' limitations, and a lack of comparability between the studies.

The studies included differed in terms of their study design. Interventions differed in terms of type of DS, DS intake pattern, training regime, time span of the intervention, and/or participant characteristics.

The studies using VD, for example, are hardly comparable because the dosage and duration of VD intake differed substantially (28, 31, 33, 34, 36). Furthermore, the participants' VD baseline levels differed between studies. For some subjects, a VD deficit was identified, while this was not the case for others. In three studies, a VD deficit among the participants was reported (28, 31, 36). This is important for the interpretation of the results, because any noteworthy effects of VD intake might be easier to achieve in case of a VD deficit. A significant positive influence of VD intake on several health variables was not determined in the study of Miller et al. (33), may be because of the participants' sufficient VD baseline levels.

All of the studies used different training programs, which might have had a different impact on study outcomes (50, 51). Thus, attention should be paid to specific types of physical exercise (for example, endurance and resistance training) for better comparability of studies and effects. Therefore, further high-quality studies with different types of physical exercise are necessary. Furthermore, the time span of the studies included, the time of each training session and the training frequency differed between the studies. A higher training intensity and longer duration of the programs are usually superior to those of a lower intensity and shorter duration (52).

The study participants' characteristics also differed considerably between the studies in terms of sex, age, time since the participants had been diagnosed with T2DM, BMI, medication, or secondary complications. This is of relevance for the recommendations the studies provide. For example, the results of Gualano et al. (30) should be interpreted with caution. The study's participants were active, their hypertension was well-controlled, and they did not have a chronic kidney disease (30). Because creatine is metabolized in the kidneys, people with chronic kidney disease may respond differently and creatine supplementation could be harmful for them.

The number of studies included in this review is small. However, one of the strengths of this review is that only (randomized) controlled trials with (placebo) control groups were included. Furthermore, the quality of the included studies was also assessed. Eight studies were assessed with 10/10 points on the PEDro scale (28–33, 35, 36), one study (37) with 7 and one study (34) with 6 points. Using the quality classification of Cashin and McAuley (27), the quality of the eight studies is rated as ‘excellent', while the quality of the two other studies is rated as ‘good’.

Studies on DS generally reveal inconsistent findings, and experts in the field are calling for well-designed high-quality studies (6). This systematic review shows that unambiguous evidence about the effects of combined DS and regular physical exercise in T2DM is also still lacking. Future research should focus on well-designed, high-quality trials. These trials should preferably include four study groups (DS-only, physical exercise-only, DS + physical exercise, no physical exercise + placebo) to statistically examine the possible additive effects of DS. Furthermore, attention must be paid to the participants' characteristics including sex, age, medication, BMI, and possible micronutrient deficits (e.g., VD deficit).

Other supplements with potential beneficial effects on T2DM variables that were evaluated in former DS only studies should be considered. For example, zinc or fiber supplementation showed positive effects on glycemic control (6).

## Conclusion

To our knowledge, this is the first systematic review investigating the possible effects of regular physical exercise in combination with DS intake on health outcomes in T2DM patients. DS intake may potentially increase the benefits of a physical training intervention for specific health outcomes in T2DM patients. However, negative effects can also be observed. Therefore, DS intake should be treated with caution when it is combined with physical training. Possible cellular and molecular mechanisms behind potential synergistic or divergent effects of exercise training and DS use in T2DM should be explored in detail in future studies for the development of safe recommendations.

## Data Availability Statement

The original contributions presented in the study are included in the article/supplementary material, further inquiries can be directed to the corresponding author/s.

## Author Contributions

EI and CB: conceptualization and writing—review and editing. MS and AL: initial literature search. FM and CB: new literature search during revision of the article. FM, EI, MO, and CB: validation and statistical analyses. FM, EI, MS, and CB: writing—original draft preparation. All authors have read and agreed to the final version of the manuscript. All authors contributed to the article and approved the submitted version.

## Funding

The IST University of Applied Sciences provided funding for the open access publication.

## Conflict of Interest

The authors declare that the research was conducted in the absence of any commercial or financial relationships that could be construed as a potential conflict of interest.

## Publisher's Note

All claims expressed in this article are solely those of the authors and do not necessarily represent those of their affiliated organizations, or those of the publisher, the editors and the reviewers. Any product that may be evaluated in this article, or claim that may be made by its manufacturer, is not guaranteed or endorsed by the publisher.
